# Pristimerin induces apoptosis and autophagy via activation of ROS/ASK1/JNK pathway in human breast cancer in vitro and in vivo

**DOI:** 10.1038/s41420-019-0208-0

**Published:** 2019-08-05

**Authors:** Qun Zhao, Yingxiang Liu, Jing Zhong, Yun Bi, Yongqiang Liu, Ziting Ren, Xiang Li, Junjun Jia, Mengting Yu, Xianjun Yu

**Affiliations:** 1 Laboratory of Inflammation and Molecular Pharmacology, School of Basic Medical Sciences & Biomedical Research Institute, Hubei University of Medicine, Shiyan, 442000 China; 2 First Clinical College, Hubei University of Medicine, Shiyan, 442000 China; 3 Hubei Key Laboratory of Natural Products Research and Development, China Three Gorges University, Yichang, 443002 China; 4 Institute of Clinical Pharmacology, Guangzhou University of Chinese Medicine, Guangzhou, 510405 China

**Keywords:** Breast cancer, Pharmacology

## Abstract

Breast cancer is the most common malignant tumor in women, and progress toward long-term survival has stagnated. Pristimerin, a natural quinonemethide triterpenoid, exhibits potential anti-tumor effects on various cancers. However, the underlying mechanism remains poorly understood. In this study, we found that pristimerin reduced the viability of breast cancer cells in vitro and the growth of xenografts in vivo, and these reductions were accompanied by thioredoxin-1 (Trx-1) inhibition and ASK1 and JNK activation. The results showed that pristimerin inhibited cell cycle progression and triggered cell apoptosis and autophagy. Furthermore, we found that the generation of reactive oxygen species (ROS) was a critical mediator in pristimerin-induced cell death. Enhanced ROS generation by pristimerin activated the ASK1/JNK signaling pathway. Inhibition of ROS with N-acetyl cysteine (NAC) significantly decreased pristimerin-induced cell death by inhibiting the phosphorylation of ASK1 and JNK. Taken together, these results suggest a critical role for the ROS/ASK1/JNK pathway in the anticancer activity of pristimerin.

## Introduction

Breast cancer is a leading cause of cancer deaths in women worldwide^1,2^. It accounts for 30% of all new cancer diagnoses and 14% of all cancer deaths in women^2^. Although the 5-year survival rate for breast cancer patients has improved due to surgery, chemotherapy and radiotherapy, severe side effects and drug resistance have become major challenges in clinical practice^3^. Therefore, the identification of novel drugs for the treatment of breast cancer is urgently needed.

Reactive oxygen species (ROS) are derived from the metabolism of oxygen and included superoxide anion radicals, singlet oxygen and hydrogen peroxide^4,5^. In resting cells, ROS are in balance with biochemical antioxidants. A moderate increase in ROS is critical for cell proliferation and differentiation, whereas excessive levels of ROS generation may result in cell death^6–8^. Accumulating evidence has suggested that chemotherapeutic agents induce cell death by enhancing ROS generation^8,9^. ROS have been demonstrated to trigger the activation of the apoptosis signal-regulating kinase 1 (ASK1)/mitogen activated protein kinase (MAPK) signaling pathway^10,11^. ASK1, a serine/threonine protein kinase, participates in cell differentiation and apoptosis^12^. Its activity is regulated during multiple processes, including dimerization, autophosphorylation, and protein-protein interactions^13^. Under nonstressed conditions, ASK1 activity was blocked upon binding to thioredoxin-1 (Trx-1)^13^. Once activated, ASK1 dissociates from Trx-1 and induces cell death by activating the c-jun-NH_2_-kinase (JNK) and p38 MAPK pathways^14^.

Pristimerin, a natural quinone methide triterpenoid extracted from various plant species in the *Celastraceae* and *Hippocrateaceae* families, may be a potential anticancer drug against various types of cancer cell lines^15–18^. In this study, we explored the effects and mechanisms of action of pristimerin on breast cancer in vitro and in vivo. We showed that pristimerin triggered G1 phase arrest, apoptosis and autophagy, and these triggered responses were mediated by ROS/ASK1/JNK signaling cascades.

## Results

### Pristimerin inhibits cell growth and induces cell cycle G1 arrest in breast cancer cells

**Fig. 1:**
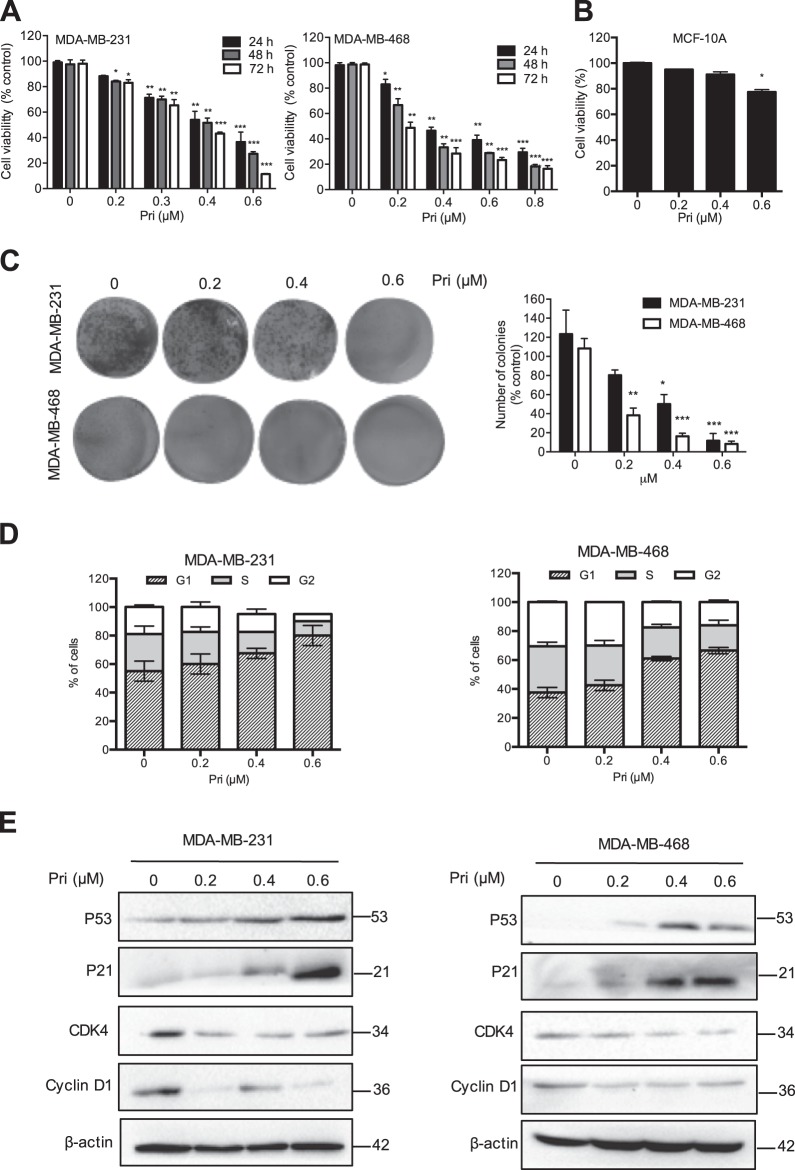
Pristimerin inhibits cell growth and induces cell cycle G1 arrest in breast cancer cells. **a** MDA-MB-231 and MDA-MB-468 cells were treated with various concentrations of pristimerin for 24, 48, and 72 h. Cell viability was analyzed by MTT assay. **b** The effect of pristimerin on normal human breast epithelial cells of the MCF-10A line is shown. **c** The results from the colony formation assay of MDA-MB-231 and MDA-MB-468 cells after pristimerin treatment are shown. **d** MDA-MB-231 and MDA-MB-468 cells were treated with pristimerin for 24 h, and the cell cycle distribution percentages were analyzed by flow cytometry. **e** The cells were treated with various concentrations of pristimerin for 24 h, and the expression of cell cycle-regulated proteins was detected by Western blotting. Results from three independent experiments are presented. *P < 0.05, **P < 0.01 and ***P < 0.001 versus control, ^#^*P* < 0.05, ^##^*P* < 0.01 and ^###^*P* < 0.001 versus pristimerin treatment

To determine the inhibitory effects of pristimerin in breast cancer cells, MDA-MB-231 and MDA-MB-468 cells were treated with various concentrations of pristimerin for 24, 48 and 72 h, and a CCK-8 assay was then performed. Pristimerin inhibited cell viability in a dose- and time-dependent manner (Fig. 1a). Interestingly, immortalized breast epithelial cells of the MCF-10A line showed strong resistance to pristimerin (Fig. 1b). Colony formation assay showed that pristimerin significantly decreased the number of colonies in both types of cells (Fig. 1c). These results suggest that pristimerin inhibits the growth of breast cancer cells in a dose- and time-dependent manner and has fewer cytotoxic effects in normal breast cells.

To test whether pristimerin inhibited cell growth by cell cycle arrest, we performed an analysis of cell distribution by cell cycle stage after pristimerin treatment. As shown in Fig. 1d, pristimerin induced the accumulation of cells in the G1 phases in MDA-MB-231 and MDA-MB-468 cells. In agreement with the cell cycle results, pristimerin significantly reduced the expression of Cyclin D1 and CDK4, and increased the levels of p53 and p21 (Fig. 1e). These data indicated that pristimerin triggered the G1 phase arrest by regulating cell cycle-related proteins.

### Pristimerin induces apoptosis and regulates apoptosis-related proteins

**Fig. 2:**
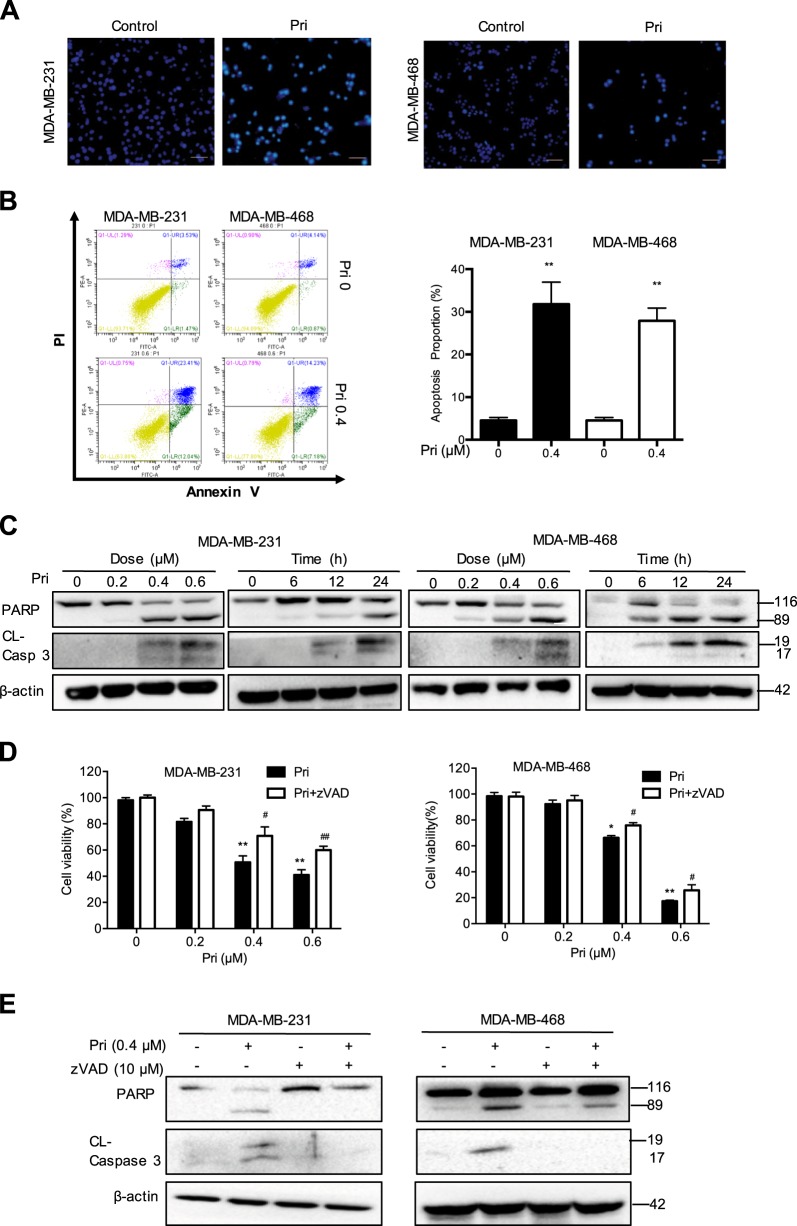
Pristimerin induces apoptosis and regulates apoptosis-related proteins. **a** MDA-MB-231 and MDA-MB-468 cells were treated with 0.4 μM pristimerin for 24 h and then stained with Hoechst 33342. The cell morphology was determined by fluorescence microscopy (×100). Bar: 100 μm. **b** The cells were treated with pristimerin (0.4 μM) for 24 h, and the percentage of apoptotic cells stained by annexin-V/PI was analyzed by flow cytometry. **c** The cells were incubated with various concentrations of pristimerin for different periods of time. The cell lysates were analyzed for PARP and cleaved of caspase-3. **d** The cells were preincubated with z-VAD (20 μM) for 2 h and then treated with pristimerin for 24 h. The cell viability was analyzed by MTT assay. **e** The cells were preincubated with z-VAD (20 μM) for 2 h and then treated with pristimerin for 24 h. The levels of PARP and cleaved caspase-3 were assessed by Western blot analysis

To explore whether pristimerin-induced cell growth inhibition is related to apoptosis, we performed Hoechst staining. Pristimerin treatment induced cell shrinkage and nuclei fragmentation (Fig. 2a). Furthermore, a significantly increased number of apoptotic cells were detected after pristimerin treatment, as determined via annexin V/PI staining (Fig. 2b). To determine the mechanism of pristimerin-induced apoptosis, we examined the expression of apoptosis-related proteins. As shown in Fig. 2c, pristimerin induced cleavage of caspase-3 and PARP in a dose- and time-dependent manner. To confirm whether caspase activation mediated pristimerin-induced cell viability loss, we used the caspase inhibitor z-VAD. We found that z-VAD partially blocked the cell viability loss induced by pristimerin (Fig. 2d). Furthermore, the cleavage of caspase-3 and PARP was also inhibited in the presence of z-VAD (Fig. 2e). Taken together, these data indicated that pristimerin induced apoptosis by caspase activation.

### Pristimerin triggers autophagy in MDA-MB-231 and MDA-MB-468 cells

**Fig. 3:**
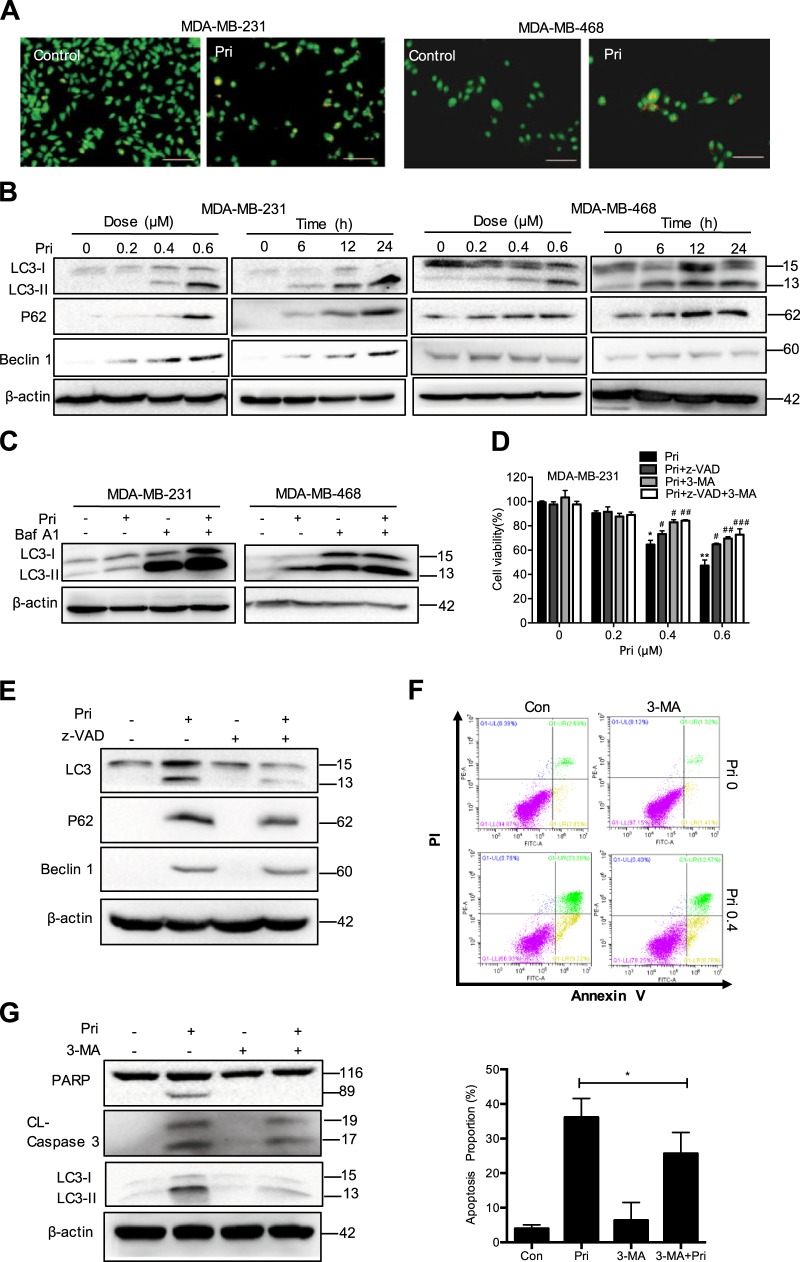
Pristimerin triggers autophagy in MDA-MB-231 and MDA-MB-468 cells. **a** MDA-MB-231 and MDA-MB-468 cells were treated with 0.4 μM pristimerin for 24 h. The acridine orange staining was assessed by fluorescence microcopy (×100). Bar: 100 μm. **b** The cells were incubated with various concentrations of pristimerin for different times. The cell lysates were analyzed for LC-3 II, p62 and Beclin-1. **c** The MDA-MB-231 cells were pretreated with 100 nM bafilomycin A1 (Baf A1) for 2 h and then incubated with 0.4 μM pristimerin for 24 h, and the levels of LC-3 were assessed by Western blotting. **d** The MDA-MB-231 cells were pretreated with z-VAD, 3-MA or combination of z-VAD and 3-MA for 2 h and then treated with 0.4 μM pristimerin for 24 h. Cell viability was analyzed by MTT assay. **e** MDA-MB-231 cells were pretreated with z-VAD for 2 h and then incubated with 0.4 μM pristimerin for 24 h, and the levels of LC-3, p62 and Beclin-1 were assessed by Western blotting. **f**, **g** MDA-MB-231 cells were pretreated with 3-MA for 2 h and then incubated with 0.4 μM pristimerin for 24 h. The cells were stained with annexin V-PE/7-AAD and analyzed by flow cytometry (**f**). The levels of PARP, cleaved of caspase-3, LC-3 II, and Beclin-1 were assessed by Western blotting (**g**). Results from three independent experiments are presented. *P < 0.05, **P < 0.01 and ***P < 0.001 versus control, ^#^*P* < 0.05, ^##^*P* < 0.01 and ^###^*P* < 0.001 versus pristimerin treatment

As cell autophagy regulates cell death, we then examined whether pristimerin could induce autophagy. Upon exposure to pristimerin, MDA-MB-231 and MDA-MB-468 cells had accumulated acidic vesicles that were observable with AO/EB staining (Fig. 3a). Furthermore, we detected the expression of several proteins that serve as markers of autophagy. Pristimerin increased the levels of LC3-II and the expression of Beclin-1 and p62 in a dose- and time-dependent manner (Fig. 3b). Bafilomycin A1 (Baf A1), an autophagy inhibitor, caused the accumulation of LC3-II by preventing autophagosomal lysosome degradation. Pristimerin also increased the accumulation of LC3-II in the presence of Baf A1 treatment (Fig. 3c). To determine the role of autophagy in pristimerin-induced cell death, we pretreated cells with the autophagy inhibitor 3-methyladenine (3-MA). The MTT assay showed that 3-MA suppressed pristimerin-induced cell viability loss (Fig. 3d). Interestingly, the combination of z-VAD and 3-MA potently blocked pristimerin-induced cell viability loss, indicating that both apoptosis and autophagy can be triggered by pristimerin (Fig. 3d).

To address the relationship between apoptosis and autophagy following pristimerin treatment, we investigated the interplay between them. First, we used the apoptosis inhibitor z-VAD to determine the role of apoptosis in autophagy induced by pristimerin. Figure 3e showed that the inhibition of apoptosis reduced the levels of LC3-II and Beclin-1, suggesting that autophagic cell death was inhibited when apoptosis was blocked. We then assessed the role of autophagy in pristimerin-induced apoptosis. Pristimerin-induced apoptosis was blocked by 3-MA (Fig. 3f). Figure 3g also showed that 3-MA impaired the cleavage of caspase-3 and PARP to a certain extent (Fig. 3g). These data suggest that inhibition of apoptosis suppresses autophagy, while autophagy contributes to apoptosis after pristimerin treatment.

### Pristimerin induces ROS generation and JNK activation

**Fig. 4:**
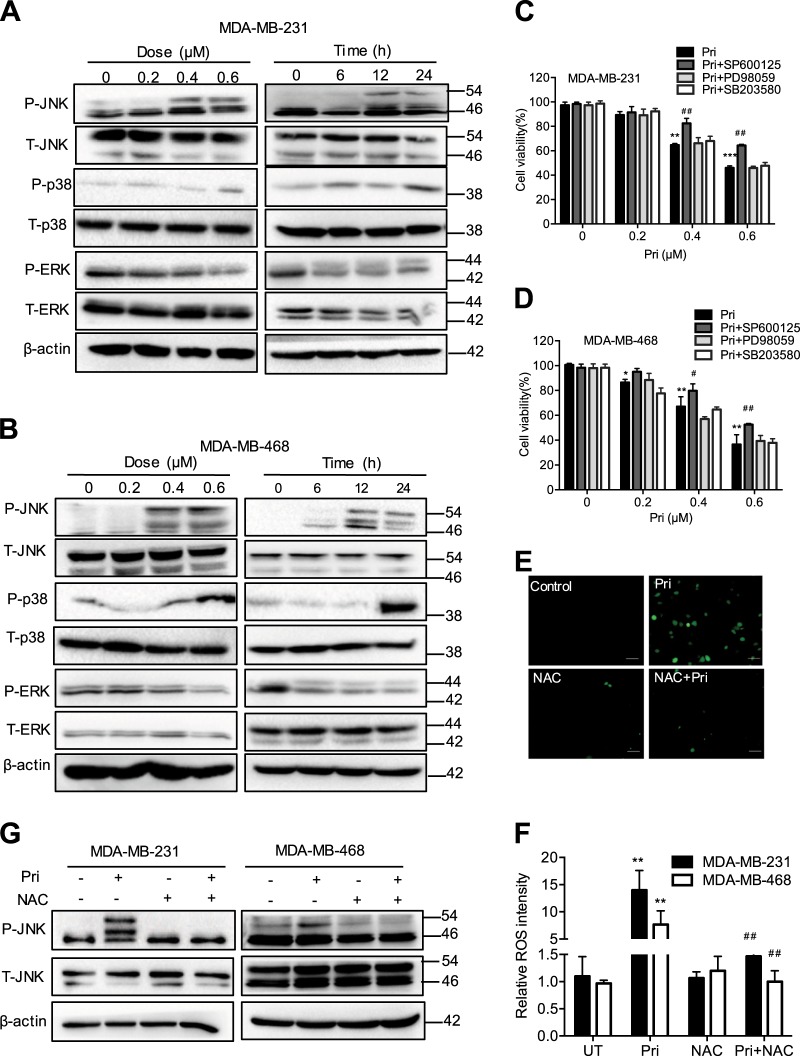
Pristimerin induces JNK activation and ROS generation. **a**, **b** MDA-MB-231 and MDA-MB-468 cells were treated with various concentrations of pristimerin for the indicated length of time. **c**, **d** The cells were preincubated with 10 μM JNK inhibitor (SP600125), ERK inhibitor (PD98059) or p38 inhibitor (SB203580) for 2 h before a 24 h pristimerin treatment. **e**, **f** MDA-MB-231 and MDA-MB-468 cells were pretreated with or without NAC (5 mM) for 1 h before a 6 h exposure to pristimerin (0.4 μM) and then treated with DCFH-DA for 30 min. Intracellular ROS generation was measured by fluorescence microscopy (×100). Bar: 100 μm. The results of the quantitative analysis of ROS are shown as histograms. (**g**) The cells were preincubated with NAC for 2 h and then treated with pristimerin for 24 h. The levels of p-JNK and JNK were analyzed by Western blotting. Results from three independent experiments are presented. *P < 0.05, **P < 0.01 and ***P < 0.001 versus control, ^#^*P* < 0.05, ^##^*P* < 0.01 and ^###^*P* < 0.001 versus pristimerin treatment

Considerable evidence has demonstrated that MAPK cascades regulate cell growth and cell death. To understand the role of MAPK activation in pristimerin-induced cell death, we showed that pristimerin treatment significantly increased the phosphorylation of JNK and p38, but phosphorylation of ERK was negligibly affected (Fig. 4a, b). Interestingly, the JNK inhibitor SP600125 significantly blocked pristimerin-induced cell viability loss, but the p38 inhibitor SB203580 did not (Fig. 4c, d). Overwhelming evidence has made clear that ROS generation regulates cell death and activates the JNK pathway. As shown in Fig. 4e, f, pristimerin triggered ROS generation in both MDA-MB-231 and MDA-MB-468 cells, and these effects were reversed by the ROS scavenger N-acetyl cysteine (NAC). In addition, pretreatment with NAC significantly inhibited the phosphorylation of JNK in both cells (Fig. 4g). These results imply that pristimerin activates the ROS/JNK signaling pathway.

### Pristimerin induces apoptosis and autophagy via the activation of ROS/JNK pathway

**Fig. 5:**
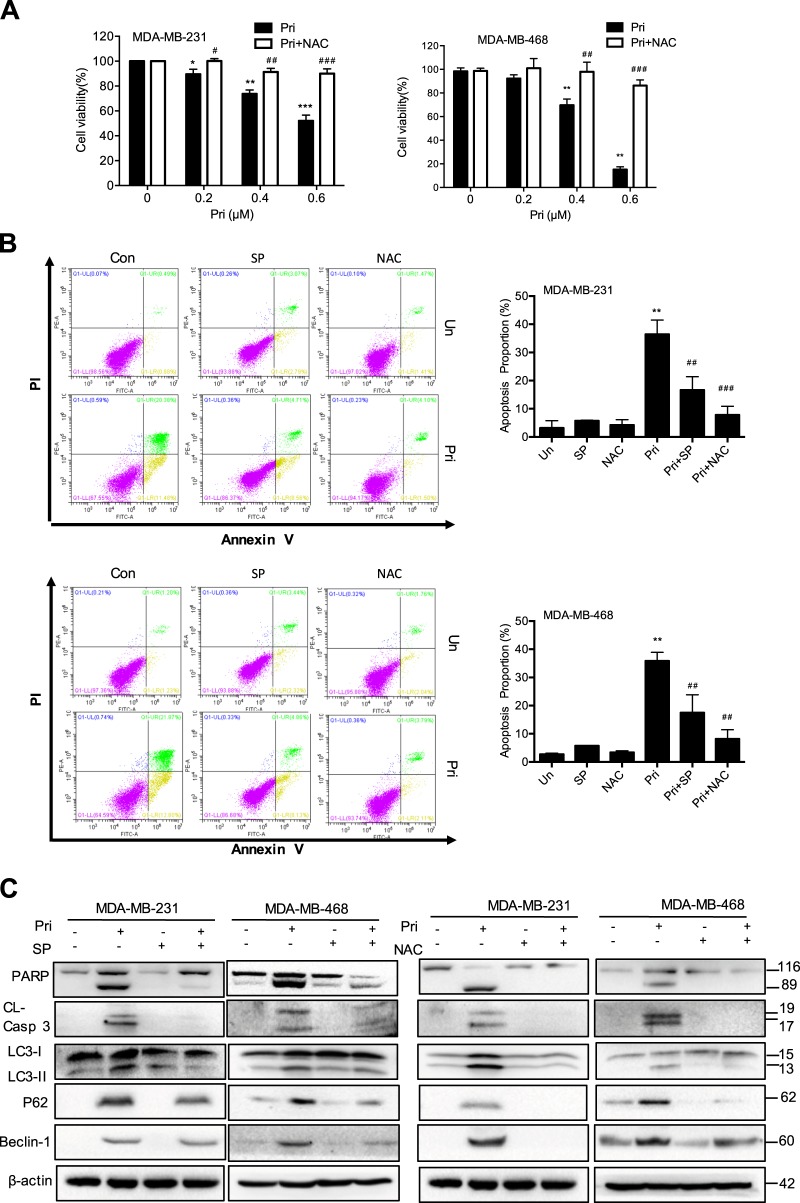
Pristimerin induces apoptosis and autophagy via the activation of ROS/JNK pathway. **a** MDA-MB-231 and MDA-MB-468 cells were preincubated with NAC for 2 h before a 24 h pristimerin treatment. Cell viability was analyzed by MTT assay. **b**, **c** MDA-MB-231 and MDA-MB-468 cells were pretreated with SP600125 or NAC for 2 h and then treated with pristimerin (0.4 μM) for 24 h. The percentage of apoptotic cells was stained by Annexin-V/PI and analyzed by flow cytometry (B). The expression of apoptosis-related proteins and the levels of LC-3 II, p62 and Beclin-1 were assessed by Western blotting (**c**). Results from three independent experiments are presented. *P < 0.05, **P < 0.01 and ***P < 0.001 versus control, ^#^*P* < 0.05, ^##^*P* < 0.01 and ^###^*P* < 0.001 versus pristimerin treatment

To further detect the role of ROS/JNK activation in pristimerin-induced cell death, we examined cell viability in the presence of NAC. NAC effectively blocked the cell viability loss induced by pristimerin and was more potent in blocking the JNK inhibitor SP600125 in MDA-MB-231 and MDA-MB-468 cells (Fig. 5a). Flow cytometric analysis showed that SP600125 and NAC could decrease pristimerin-induced apoptosis (Fig. 5b). Western blot analysis revealed that SP600125 and NAC reversed pristimerin-induced activation of apoptosis-related proteins (Fig. 5c). In addition, both SP600125 and NAC significantly blocked the levels of LC3-II, p62 and Beclin-1 expression (Fig. 5d). These results reveal that cell apoptosis and autophagy provoked by pristimerin are associated with JNK activation and ROS generation.

### Pristimerin induces activation of ASK1

**Fig. 6:**
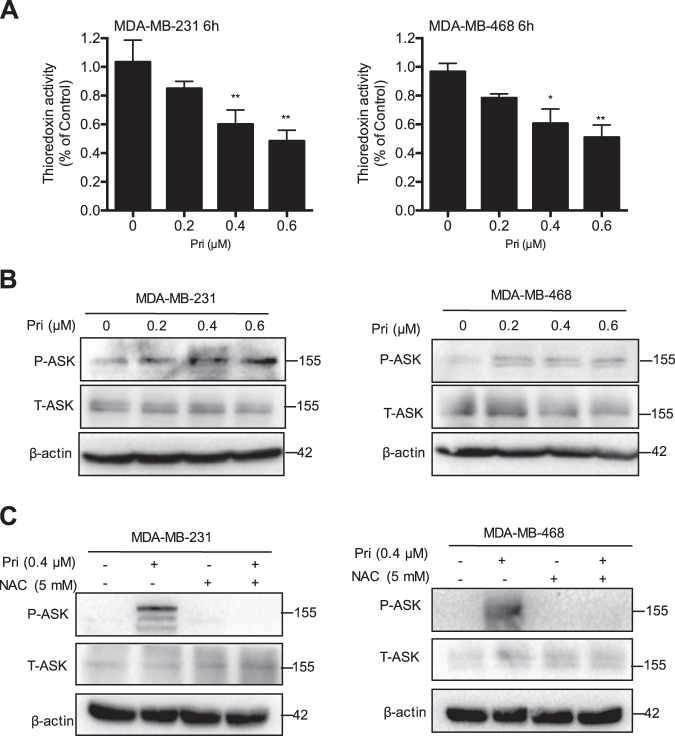
Pristimerin induces activation of ASK1. **a** MDA-MB-231 and MDA-MB-468 cells were treated with pristimerin at the indicated concentrations for 6 h, and Trx-1 activity in the cell lysates was determined by insulin endpoint assay. **b** Cells were pretreated with the indicated concentrations of pristimerin for 24 h. The expression of p-ASK1 at threonine 845, ASK1 was determined by Western blotting. **c** The cells were pretreated with NAC for 2 h and then treated with pristimerin (0.4 μM) for 24 h. The expression of p-ASK1 and ASK1 was assessed by Western blot. Results from three independent experiments are presented. *P < 0.05, **P < 0.01 and ***P < 0.001 versus control, ^#^*P* < 0.05, ^##^*P* < 0.01 and ^###^*P* < 0.001 versus pristimerin treatment

ASK1 is known as a member of the MAPK kinase kinase. Under ROS stress, ASK1 is activated and dissociated from Trx-1. We found that pristimerin suppressed Trx-1 activity in a dose-dependent manner (Fig. 6a). To address the activation of ASK1, we examined the phosphorylation of the Thr 845 residue of ASK1. As shown in Fig. 6b, pristimerin treatment resulted in a significant increase in ASK1, resulting in a significant increase in phosphorylation in MDA-MB-231 and MDA-MB-468 cells. ROS activate the ASK1/JNK signaling pathway. Our results demonstrated that NAC effectively inhibited pristimerin-triggered ASK1 activation (Fig. 6c). These results suggest that activation of the ROS/ASK1 pathway is required for pristimerin-induced cell death.

### In vivo antitumor efficacy of pristimerin in a xenograft mouse model

**Fig. 7:**
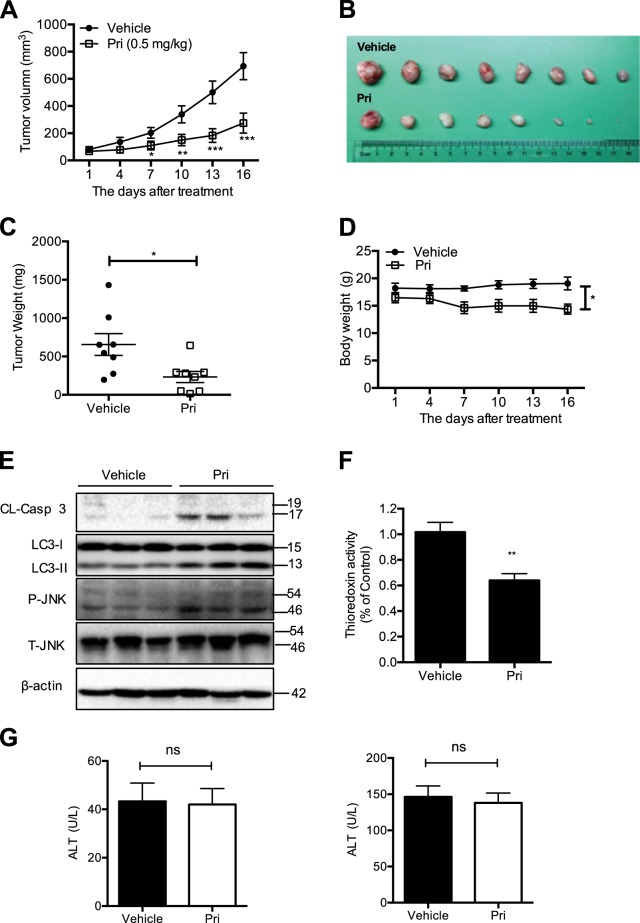
In vivo antitumor efficacy of pristimerin in a xenograft mouse model. **a** MDA-MB-231 cells were subcutaneously injected into the nude mice. Intraperitoneal treatment of the vehicle or of 0.5 mg/kg pristimerin (*n* = 8) daily was administered. The tumor volume was monitored. **b** At the end of the experiment, the tumors were excised. Images of resected human breast tumor taken for the vehicle and pristimerin treatment groups. **c** The tumors were weighed. **d** The body weights were calculated for the vehicle and pristimerin treat mice during the experiment. **e** Western blot analysis was used to determine the expression of cleaved caspase-3, LC-3 II, p-JNK, and JNK from respective tumor tissue lysates. **f** The Trx-1 activity was assessed in tumor tissue lysates. **g** Nude mice were treated with pristimerin by intraperitoneal injection every day for 14 days, and blood samples were collected and measured for ALT and AST expression in the vehicle and pristimerin-treatment mice. Results from three independent experiments are presented. *P < 0.05, **P < 0.01 and ***P < 0.001 versus control, ^#^*P* < 0.05, ^##^*P* < 0.01 and ^###^*P* < 0.001 versus pristimerin treatment

To further evaluate the activity of pristimerin in vivo, we investigated its ability to suppress the growth of MDA-MB-231 tumor xenografts in nude mice. As shown in Fig. 7a–c, pristimerin significantly suppressed tumor volume and weight in the mice. However, pristimerin treatment caused milder weight loss (Fig. 7d). Western blot analysis of the tumor tissues showed that pristimerin increased the levels of cleaved caspase-3, LC-3 II and phosphorylation-JNK (Fig. 7e). In addition, pristimerin inhibited the activity of Trx-1 in the tumors, a finding consistent with observations in vitro (Fig. 7f). To explore the potential cytotoxic effects of pristimerin on normal tissues, we compared ALT and AST expression in pristimerin-treated and vehicle mouse groups and found no significant differences (Fig. 7g). These data show that pristimerin inhibits the growth of breast cancer in vivo.

## Discussion

**Fig. 8:**
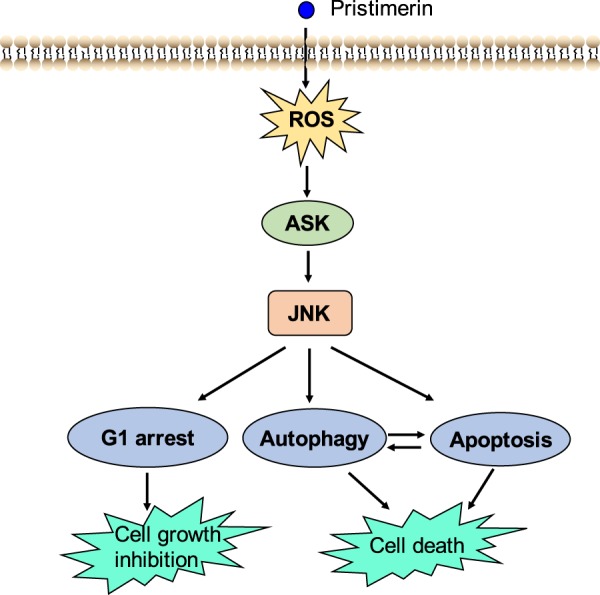
Scheme of pathway induced by pristimerin

Previous studies have demonstrated that pristimerin has various bioactivities, including anti-inflammatory, antioxidative and anticancer effects^15–18^. In this study, we demonstrated that pristimerin exhibited potent antitumor activity against breast cancer in vitro and in vivo. Mechanistically, pristimerin attenuated Trx-1 activity, leading to ROS accumulation. Overproduction of ROS induced by pristimerin resulted in ASK1/JNK activation, which caused cell apoptosis and autophagy (Fig. 8).

Apoptosis is involved in the development, maintenance and tissue homeostasis of multicellular organisms^19^. Moreover, apoptosis is associated with tumor formation and cancer therapy^20,21^. The extrinsic and intrinsic apoptotic pathways are two common ways that initiator caspases are activated, that in turn, proteolytically cleave and activate effector caspase-3 and caspase-7, resulting in cleavage of downstream substrates, such as PARP^22–24^. In the present of study, the results showed that pristimerin induced apoptosis of breast cancer cells in vitro and in vivo (Figs. 2 and 7). Surprisingly, the caspase inhibitor z-VAD could not completely prevent cell death, leading us to explore other caspase-independent pathways (Fig. 2d). Autophagy, an important physiologic process associated with cell proliferation, survival, tumorigenesis, development and stress responses, can be leveraged in a novel strategy for increasing the antitumor efficacy of chemotherapy compounds^25–27^. Interestingly, the autophagy inhibitor 3-MA blocked pristimerin-induced cell viability loss and apoptosis (Fig. 3d–f). Accordingly, we found that enhanced LC-3 II was induced by pristimerin treatment in vitro and in vivo (Figs. 3 and 7). Considerable evidence has indicated that the relationship between apoptosis and autophagy is complex and that it can be cooperative or antagonistic^28,29^. Herein, inhibition of autophagy by 3-MA suppressed pristimerin-induced apoptosis, indicating that autophagy contributes to apoptosis. Moreover, z-VAD decreased the expression of LC-3 II, implying that inhibition of apoptosis impaired autophagy (Fig. 3e, f). The mechanism of connection between apoptosis and autophagy needs to be clarified in future work.

ROS are known as important upstream molecules in the progression of cell death and survival. The level of ROS plays a key role in cell survival and apoptosis, whereas excessive ROS can induce cell damage^30^. Recently, numerous ROS-inducing agents have been reported to selectively kill cancer cells without inducing significant toxicity in normal cells^6,9,31,32^. In this study, we found that pristimerin significantly induced ROS generation ~10-fold at concentrations of 0.4 μM compared with untreated cells (Fig. 4e, f). Antioxidants NAC markedly abolished pristimerin-induced cell viability loss, ROS generation, autophagy and apoptosis (Fig. 5). Therefore, ROS generation may play a critical role in pristimerin-mediated antitumor activity, which implies that the effect of pristimerin may be associated with cell redox system imbalance.

Trx-1, a major redox protein in cytoplasm, is a critical antioxidant protein that regulates a wide range of cell processes^33–35^. Elevated ROS generation can be triggered by Trx-1 inhibition or by changing its redox state from the reduced to the oxidized state, which results in apoptosis. Indeed, pristimerin effectively inhibited Trx-1 activity in breast cancer cells in vitro and in vivo (Figs. 6a and 7f). The reduced form of Trx-1 binds to the N-terminal region of ASK1 and inhibits its kinase function in a normal state. However, ASK1 dissociates from Trx-1 and is activated after Trx-1 is converted to the oxidized form, leading to cell death by activation of the JNK and p38 pathways^14^. Our study indicated that pristimerin was involved in the release of ASK1 from Trx-1, which resulted in phosphorylation of ASK1 at Thr 845 (Fig. 6b). Moreover, pristimerin-induced ASK1 activation was inhibited by the ROS scavenger NAC (Fig. 6c). These results suggest that ROS and ASK1 play an important role in pristimerin-induced cell death.

A range of studies have revealed that MAPKs are mediators of cellular responses to extracellular signals. JNK has been shown to be an essential mediator in cell death that is induced by various anticancer drugs^36–38^. We found that pristimerin induced a significant increase in JNK phosphorylation (Fig. 4a, b). We also revealed that pristimerin-induced cell death was markedly restored by the JNK inhibitor SP600125, indicating that the JNK activation induced by pristimerin contributed to cell death (Fig. 4c, d). To explore whether ROS accumulation affects JNK signaling, the effects of pristimerin on these proteins were examined. The ROS scavenger NAC completely blocked pristimerin-induced apoptosis and autophagy, while the JNK inhibitor SP600125 partially abolished these effects (Fig. 5). These data suggest that pristimerin induces cell death through the ROS/JNK signaling pathway.

In conclusion, our study demonstrated the antitumor effects of pristimerin on breast cancer in vitro and in vivo and uncovered the potential molecular mechanisms. We found that pristimerin induced G1 cell cycle arrest and caused cell apoptosis and autophagy. Furthermore, the results showed that the Trx-1/ROS/JNK and Trx-1/ASK1/JNK pathways played important roles in pristimerin-induced cell death. We also demonstrated that pristimerin markedly suppressed tumor growth in mice bearing xenografts without obvious acute toxic effects. Therefore, this study indicates that pristimerin is a great potential drug in the treatment of breast cancer.

## Materials and methods

### Reagents

Purified pristimerin (>98%) was purchased from the Shanghai Yuanye Biotech Company (Shanghai, China). N-acetyl-L-cysteine (NAC) and 3-MA were purchased from Sigma (St. Louis, MO, USA). z-VAD(OMe)-FMK (z-VAD), JNK inhibitor (SP600125) and p-38 inhibitor (SB203580) were obtained from MCE (Shanghai, China). Antibodies were used against PARP, caspase-3, Cyclin D1, Cdk4, p21, Atg-7, p62, JNK, phospho-JNK, p38, phospho-p38, ERK, phospho-ERK, ASK1, phospho-ASK1 (Thr845) (Cell Signaling Technology, Beverly, MA, USA) and β-actin (Sigma, St. Louis, MO, USA).

### Cell line and culture

Human breast cancer cell lines (MDA-MB-231 and MDA-MB-468) and normal breast epithelial cells (MCF-10A) were provided by Prof. Tao Zhu at the University of of Science and Technology of China, Hefei, Anhui, China^39^. All cells in this study were cultured as recommended by ATCC.

### Cell viability assay

Cells (5 × 10^4^ cells/well) were seeded in 96-well microplates and cultured for 24 h and then treated with the indicated compounds for the indicated periods. Cell viability was determined by MTT assay^40^.

### Cell cycle analysis by flow cytometry

Cells (5 × 10^5^ cells/well) were seeded in a 6-well microplate and synchronized and then treated with the indicated concentrations of pristimerin for 24 h. The cells were collected and washed in PBS, fixed in 70% ethanol and maintained at 4 °C overnight. The cells were centrifuged and washed with PBS containing 1% FBS, followed by treatment with RNaseA for 15 min at 37 °C and then by propidium iodide staining. The cell analysis was performed by flow cytometry.

### Hoechst 33342 and cell apoptosis assay

Cells (5 × 10^5^ cells/well) were seeded and then treated with various concentrations of pristimerin for 24 h. After incubation, the cells were fixed with 4% paraformaldehyde for 30 min and then stained with a Hoechst 33342 solution for 20 min in the dark at 37 °C. The cells were examined by fluorescence microscopy for morphological changes. In addition, the cells were resuspended in 1× binding buffer and then incubated with annexin V and PI for 30 min in the dark at room temperature, and the number of apoptotic cells was determined by flow cytometry.

### Measurement of ROS

Cells (2 × 10^5^ cells/well) were plated in 12-well plates and treated with pristimerin in the absence or presence of NAC. The cells were then incubated with DCFH-DA (10 μM) in DMEM without FBS for 30 min at 37 °C. The cells were washed three times, and the level of ROS was determined by fluorescence microscopy (Leica, Wetzlar, Germany).

### Western blotting

Cells were cultured and then treated with the indicated concentrations of pristimerin for different lengths of time. Cells were washed with PBS and harvested and then lysed in RIPA buffer containing a protease and phosphatase inhibitor cocktail for 30 min on ice. After centrifugation, the supernatant protein was collected and quantified using a BCA assay. Equal amounts of proteins were subjected to SDS-polyacrylamide gel electrophoresis at 100 V for 2 h and transferred to PVDF membranes (Millipore, Plano, TX, USA). After blocking with 5% nonfat milk, the membranes were incubated with primary antibodies at 4 °C overnight, and the membrane-bound antibodies were visualized using peroxidase-conjugated secondary antibodies. Specific antibody binding was detected by a chemiluminescence kit (Millipore, Plano, TX, USA).

### Tumor xenograft in nude mice

Female BALB/c-nu mice (Beijing HuaFuKang Bioscience Co., Ltd, Beijing, China) were purchased at 4 weeks of age and housed in a specific pathogen-free environment under a 12-h light and 12-h dark cycle with free access to water and food. A total of 1 × 10^6^ MDA-MB-231 cells in 100 μl PBS were subcutaneously injected into the right flank of each mouse. The tumors were macroscopic after 1 week, and at that time, the mice were randomly divided into two groups: a vehicle group and a pristimerin group (eight mice in each group). The vehicle group received an intraperitoneal injection of 100 μl 5% DMSO every other day, while the pristimerin group was injected with 100 μl pristimerin diluted with 5% DMSO (0.5 mg/kg). The tumor size was measured using slide calipers, and the tumor volume was calculated as 0.5 × a × b^2^, where a is the length of the tumor, and b is the width. On the day of the final treatment, the mice were euthanized, and the tumors were removed, weighed and frozen immediately in liquid nitrogen for subsequent Western blotting.

### Statistical analysis

All data represent at least three independent experiments and are expressed as the mean ± standard deviation unless otherwise noted. Statistical comparisons were made using one-way ANOVA. *P* *<* 0.05 was considered to represent a statistically significant difference.

### Ethical statement

All animal experiments were performed in accordance with guidelines for animal treatment of Hubei University of Medicine. All experimental protocols in our study were approved by the Ethics Committee of Hubei University of Medicine.
